# How Old Is Your Brain? Slow-Wave Activity in Non-rapid-eye-movement Sleep as a Marker of Brain Rejuvenation After Long-Term Exercise in Mice

**DOI:** 10.3389/fnagi.2018.00233

**Published:** 2018-08-07

**Authors:** Maria Panagiotou, Kostas Papagiannopoulos, Jos H. T. Rohling, Johanna H. Meijer, Tom Deboer

**Affiliations:** ^1^Laboratory for Neurophysiology, Department of Cell and Chemical Biology, Leiden University Medical Center, Leiden, Netherlands; ^2^Department of Digital Security, Radboud University, Nijmegen, Netherlands

**Keywords:** EEG, SWA, spectral analysis, running wheel, exercise, aging

## Abstract

Physical activity is beneficial for health. It has been shown to improve brain functioning and cognition, reduce severity of mood disorders, as well as facilitate healthy sleep and healthy aging. Sleep has been studied in healthy aged mice and absolute slow-wave-activity levels (SWA, electroencephalogram power between 0.75 and 4.0 Hz) in non-rapid-eye-movement sleep (NREM) were elevated, suggesting changes in brain connectivity. To investigate whether physical activity can diminish this aging-induced effect, mice of three age groups were provided with a running wheel (RW) for 1–3 months (6-months-old, *n* = 9; 18-months-old, *n* = 9; 24–months-old, *n* = 8) and were compared with control sedentary mice (*n* = 11, *n* = 8 and *n* = 9 respectively). Two weeks before the sleep-wake recordings the running wheels were removed. The electroencephalogram (EEG) and electromyogram were continuously recorded during undisturbed 24 h baseline (BL) and a sleep-deprivation was conducted during the first 6 h of the second day. Increased waking and decreased NREM sleep was found in the young RW mice, compared to young controls. These effects were not evident in the 18 and 24 months old mice. Unlike sleep architecture, we found that SWA was altered throughout the whole age spectrum. Notably, SWA was increased with aging and attenuated with exercise, exhibiting the lowest levels in the young RW mice. To utilize the cross-age revealing features of SWA, we applied machine learning techniques and found that characteristic information regarding age and exercise was enclosed in SWA. In addition, with cluster analysis, we could classify and accurately distinguish the different groups based solely on their SWA. Therefore, our study comprises a three-fold contribution: (a) effects of exercise on sleep are sustained following 2 weeks after removal of the wheel, (b) we show that EEG SWA can be used as a physiological marker of brain age in the mouse, (c) long-term voluntary regular age-matched exercise leads to a younger phenotype.

## Introduction

Sleep is a vital physiological routine that has a critical function in promoting the health of all living organisms. It is regulated by two processes, the circadian and homeostatic process (Borbély et al., 2016; Achermann and Borbely, 2017). The circadian process is controlled by the circadian pacemaker, which is located in the suprachiasmatic nucleus and fluctuates with a cycle of approximately 24 h, promoting wakefulness during the daily active phase and sleep during the rest phase. The homeostatic process refers to the sleep drive that builds-up during wakefulness and is dissipated during sleep. In mammals, sleep homeostasis is thought to be reflected in electroencephalographic slow-wave activity during non-rapid-eye-movement (NREM) sleep (Achermann and Borbely, 2017).

In healthy aged humans, several sleep parameters deteriorate and the overall sleep quality is impaired (Landolt et al., 1996; Carrier et al., 2001; Crowley, 2011). To further elucidate this disruption, animal models have been used to investigate sleep in the course of aging (Welsh et al., 1986; Colas et al., 2005; Farajnia et al., 2012; Hasan et al., 2012; Banks et al., 2015; Panagiotou et al., 2017; McKillop et al., 2018). It was recently demonstrated that slow-wave activity in the NREM sleep electroencephalogram (EEG SWA) was substantially increased in aged, as compared to young mice. In addition to the increased SWA levels, the overall morphology of the slow-waves was shown to be changed, suggesting cortical brain alterations (Panagiotou et al., 2017).

Sleep disorders, commonly encountered in aging, can be rectified with the use of pharmacological remedies (Kamel and Gammack, 2006; Tariq and Pulisetty, 2008). Although pharmacology often comprises the first-order treatment, lifestyle interventions such as exercise, have been proposed as a potential to ameliorate sleep disturbances (Chennaoui et al., 2015). Regular physical activity is, generally, considered to be beneficial for health (Booth et al., 2012). Aerobic exercise, such as running, has been demonstrated to have a role in brain function and cognition, in preventing and treating or reducing severity of stress-related mood disorders, as well as in promoting healthy sleep cycles and healthy aging (Hillman et al., 2008; Kaliman et al., 2011; Hötting and Röder, 2013). The effect of regular physical activity in parameters such as cognitive functioning and performance in elderly people, as well as aged animals, has been thoroughly investigated and found positive (Heyn et al., 2004; Voss et al., 2013b). However, the long-term impact of regular exercise on sleep and EEG SWA levels remains largely uncharted in the course of aging.

Concretely, acute and chronic effects of exercise on sleep have been investigated in young humans (Kredlow et al., 2015). However, detailed studies in elderly humans are scarce and mainly demonstrate a more moderate effect of regular exercise on sleep quality compared to young subjects (Yang et al., 2012; Kredlow et al., 2015; Dolezal et al., 2017). In rodent-based animal models, analogous findings are also limited. Access to exercise equipment, such as running wheels, as an equivalent to treading mills in humans, has shown beneficial effects on sleep in young mice (Lancel et al., 2003), and young and aged F344 rats (Blanco-Centurion and Shiromani, 2006).

In the current study, we investigated the effect of regular physical activity on sleep architecture and EEG SWA of male C57BL/6J mice of three different age groups (6, 18, and 24 months old). For this, a running wheel was introduced in the cage of the mice that could be used voluntarily on a daily basis for 1–3 months. The wheels were permanently removed 2 weeks prior to the sleep recordings, therefore, a sustained effect of physical activity on sleep was profoundly studied. Based on our previous results, showing a lower daily amplitude in sleep-wake distribution and an increase in SWA in NREM sleep in the course of aging (Panagiotou et al., 2017), we hypothesized a beneficial effect following long-term exercise consisting of a sleep pattern amelioration and lower SWA in NREM sleep in these mice.

## Materials and Methods

### Animals

**FIGURE 1:**
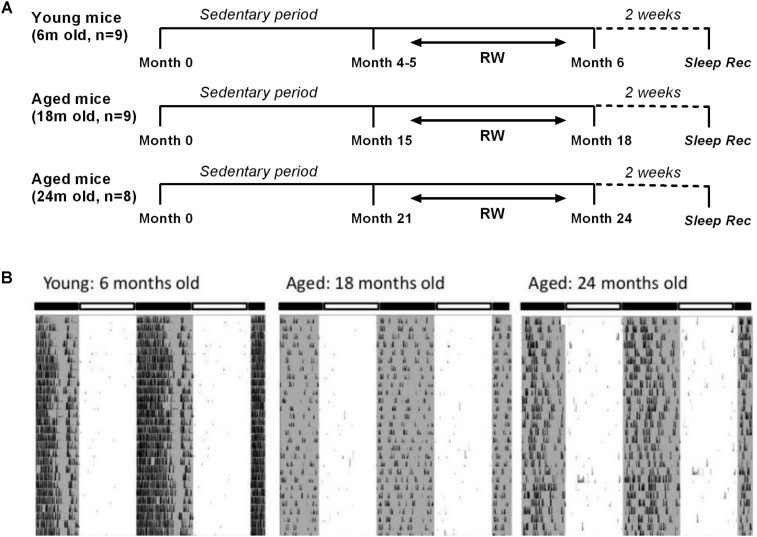
**(A)** Schematic overview of the experimental design. Mice were provided with a running wheel in their cage for long-term daily voluntary use of approximately 1 month (young group) and 3 (18 and 24 months old groups) prior to the sleep recordings. *(RW: running wheel; Sleep rec: sleep recordings).* **(B)** Representative double-plotted actograms of 28 days running wheel activity from a 6-month, an 18-month and a 24-month old mouse. Young mice showed an increased average daily running wheel activity as compared to aged mice (*6 months old:* 27746 ± 1974 counts/24 h, *18 months old:* 5190 ± 2521 counts/24 h, *24-months old:* 6458 ± 1884 counts/24 h) (*post hoc t*-tests with Bonferroni multiple comparisons correction, *p* < 0.05 after significant ANOVA, main effect ‘exercise’).

Male C57BL/6JOlaHsd mice of three age groups (6, 18, and 24 months old, *n* = 54) (Harlan, Horst, Netherlands) were used for this study. They were partitioned into six distinct groups. Three control groups in which mice were sedentary throughout their life (*n* = 11, 8, and 9 with increasing age respectively) and three running-wheel (RW) groups where a running wheel was introduced in the cage (*n* = 9, 9 and 8 with increasing age respectively), for voluntary use in a daily basis in order for the mice to be exposed to a type of aerobic exercise as preferred in human studies. Aerobic exercise, also characterized as endurance activity or cardiovascular exercise, consists of a sustained period of rhythmic movement of large muscles, in which pumping of oxygenated blood by the heart is required so as oxygen is delivered to the muscles to generate energy. A beneficial effect of specifically aerobic fitness training on brain function and cognition has been explored (Hillman et al., 2008). In particular, for the 6 months old the access lasted for approximately a period of 1 month, and for the two aged groups for at least 3 months, prior to the sleep recordings. Therefore, young mice had access to the running wheel from the age of 4–5 months, while 18 and 24 months old mice had access to the running wheel from the age of 15 and 21 months respectively, prior to the sleep recordings (**Figure 1A**). Throughout the text we often refer to the running wheel activity as ‘exercise’. The mice were individually housed under controlled conditions (12:12 h light:dark cycle; lights on at 10:00) with food and water *ad libitum* in a temperature controlled room (21–22°C).

All animal experiments were approved by the Animal Experiments Ethical Committee of the Leiden University Medical Center (Netherlands) and were carried out in accordance with the EU Directive 2010/63/EU on the protection of animals used for scientific purposes.

### Behavioral Activity Recordings

The voluntary RW animals were individually housed in cages with a running wheel, to record their behavioral activity pattern. The number of wheel revolutions was digitized in 1 min bins with a computerized recording system (Clocklab; Actimetrics). Behavioral activity was plotted in actograms, and the circadian period, the amount of wheel running per 24-h, as well as the strength of the circadian rhythm, defined as the difference between the 95% confidence limit and the peak in the periodogram, was calculated with F-periodogram analysis, as described previously (Jenni et al., 2006; Stenvers et al., 2016), using the Clocklab analysis software (Actimetrics, Wilmette, IL, United States). Control animals, without a wheel, were housed individually in similar cages, for the same duration.

### Surgeries

On the day of the surgery, the running wheel was permanently removed from the cages of the animals. Before the surgeries, 6, 18, and 24 month old RW mice weighed on average 24.4, 36.9, and 38.5 g respectively and 6, 18, and 24 month old control mice 30.7, 33.5, 32.7 g respectively. Under deep anesthesia (Ketamine 100 mg/kg; Xylazine 10 mg/kg; Atropine 1 mg/kg) EEG recording screws and electromyogram (EMG) electrodes (Plastics One) were implanted as described previously (Deboer et al., 2013; Panagiotou et al., 2017). One EEG electrode was placed over the right hemisphere (2 mm lateral to the midline of the skull, 2 mm posterior to bregma) above the somatosensory cortex and the other was placed above the cerebellum (midline, 2 mm caudal to the lambda) as a reference. EMG electrodes were placed on the neck muscle. The wire branches of all electrodes were set in a plastic pedestal (Plastics One, Roanoke, VA, United States) fixed to the skull with dental cement. The mice were allowed to recover for 7–10 days.

### EEG Recordings

The EEG and EMG were recorded with a portable recording system (PS 1 system, Institute of Pharmacology and Toxicology, Zurich, Switzerland) as previously described (Deboer et al., 2007, 2013; Panagiotou et al., 2017). Before each recording, a calibration signal was recorded on the EEG and EMG channels. Both signals were amplified, conditioned by analog filters and sampled at 512 Hz. The signals were filtered through a digital finite impulse response filter and stored with a resolution of 128 Hz. EEG spectra were computed for 4-s epochs.

To record the EEG and EMG, animals were placed into experimental chambers and connected through a flexible cable and a counterbalanced swivel system to the recording setup. Conditions in the experimental chamber were similar to the home cage, without running wheel availability. Before starting the experiment, the animals were allowed to adjust to the experimental conditions for a week. Subsequently, a baseline (BL) day was recorded, starting at lights on. At the start of the second day, 6 h of sleep-deprivation (SD) were conducted by gentle handling (Huber et al., 2000; Deboer et al., 2013; Panagiotou et al., 2017). During that period, every time the animals appeared drowsy, or the EEG exhibited slow waves, the animals were mildly disturbed by noise, movement of bedding, or introducing new nesting material or food into the cage. EEG and EMG were recorded continuously during SD and, subsequently, for 18 h to investigate sleep characteristics after SD. The time between the day of the surgery and the BL sleep recordings was approximately 2 weeks. All animals contributed once to the sleep recordings and no animals were double used.

### Data Analysis and Statistics

Three vigilance states (Waking, NREM sleep and REM sleep) were scored offline in 4 s epochs by visual inspection of the EEG and EMG signals as well as EEG power in the slow-wave range, as described previously (Huber et al., 2000; Deboer et al., 2013; Panagiotou et al., 2017). For each epoch, the EEG power in the delta (0.75–4.0 Hz) and theta band (6.25–9.0 Hz) and the integrated EMG value were graphically displayed on a PC monitor to enable scoring of the different vigilance states (waking, NREM sleep and REM sleep). The vigilance states were expressed as a percentage of artifact-free recording time. Epochs with artifacts were excluded from the subsequent data analysis of the power spectra, but vigilance states could always be determined. Spectral analysis was performed using a fast Fourier transform (FFT; 0.5–25 Hz). Analysis of variance (ANOVA) with factors ‘exercise’ or ‘strength’ was performed to detect differences in daily running wheel activity amount and strength of the 24-h rhythm respectively across the age groups. Three-way ANOVA with factors ‘treatment,’ ‘time of day,’ ‘day’ for 2-h vigilance states and EEG SWA and two-way ANOVA with factors ‘treatment’ and ‘Light-dark’ for 12-h vigilance states and ‘treatment’ and ‘frequency bin’ for EEG spectra was applied to test the differences between the groups and experimental conditions. One-way ANOVA factor ‘group’ was applied to detect differences across six groups in EEG SWA in 12 h BL light period. Additionally, to test whether SD induced alterations in EEG SWA, for each group separately a two-way ANOVA was performed with factors ‘time of day’ and ‘day.’ When appropriate, paired and unpaired *post hoc* Bonferroni-corrected student’s *t*-tests were applied to determine the effects of exercise (treatment) or SD.

### Profiling and Classifying Slow-Wave Activity

We performed additional statistical analysis on the SWA data. First, we used a supervised learning approach to examine if the SWA data contained sufficient information to correctly classify the different groups based on their age and the availability of the running wheel. Next, an unsupervised learning approach was taken to identify, in an unbiased fashion, clusters from the data. The distances between the central points of the identified clusters (centroids) show the similarity of the SWA between clusters; a larger distance signifies a larger difference in SWA. For our analysis, we used SWA values, averaged over 1-h intervals, obtained during the 12 h BL light period, resulting in a 12-dimensional signal for every mouse. Matlab was used for the following analysis (The MathWorks, Inc., Natick, MA, United States).

#### Supervised Learning Approach

We examined whether the data convey information about the age and the exercise of the mice using the generic perceived information (PI) metric (Renauld et al., 2011; Durvaux et al., 2014). To acquire this metric we used profiling techniques (supervised statistical machine learning) that originate from signal processing (Van Trees, 1992) and have been successfully used for classification purposes in the context of EEG brain activity (Lange et al., 2017). Subsequently, we tested whether this available information could be concretely used in order to find the previously unknown age and exercise level of a mouse. The effectiveness of this classification stage was quantified using the stage’s success rate metric.

Throughout this section, capital letters denote random variables, small case letters denote their instances and calligraphic letters denote sets. Bold font denotes vectors and matrices. The slow wave activity is denoted as random variable *S* over alphabet 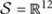 and a particular SWA signal as *s*. Similarly, we describe age with random variable *A* over 𝒜 = {6,18,24} months and exercise with random variable *E* over ε = {*yes, no*}. We, also, define the age/exercise class with random variable *C* = (*A,E*) over all possible age/exercise pairs *C*, e.g., the 6-month-old mice that exercise are described by class *c* = (6,*yes*).

We grouped together the SWA signals of all mice that belong to a certain age/exercise class *c*, referring to it as the dataset 𝒟 with respect to class *c*, or 𝒟^c^ for short. The grouping process was repeated for all *c* ∈ 𝒞. Our supervised approach required separate datasets for profiling and testing, thus we partitioned 𝒟^c^ to ${𝒟}_{p}^{c}$ and ${𝒟}_{t}^{c}$, denoting the profile subset and the test subset respectively.

The profiling step of the classification technique uses the profiling dataset ${𝒟}_{p}^{c}$ to estimate a probabilistic model that describes the behavior of class *c*. Specifically, we assumed that for a given age/exercise class *c*, SWA follows a multivariate normal distribution, i.e., Pr(*S* = *s*|*C* = *c*) ∼ *N*(μ_c_, ∑_c_). We used ${𝒟}_{p}^{c}$ to estimate the mean μ_c_ and the covariance matrix ∑_c_ of the distribution, resulting in the probabilistic model P^r⁢ model(S =s|C =c), also known as the *template* of *c*. Note that prior to parameter estimation, we performed dimensionality reduction on ${𝒟}_{p}^{c}$ via principal component analysis (PCA) on the SWA signals in order to extract most of the available information, while preventing the model training process from becoming too data intensive.

The test step of the classification technique uses the SWA signals of test dataset ${𝒟}_{t}^{c}$. Computing the PI metric requires approximating the true and unknown distribution *Pr*_true_(S=s—C=c), which can be sampled from ${𝒟}_{t}^{c}$. Computing the success rate metric requires comparing all signals in ${𝒟}_{t}^{c}$ against all class templates and finding the best-matching one.

#### Perceived Information

The PI can quantify the amount of information that the SWA S contains about the class *C*, taking into account the divergence between the real and the estimated models. Rephrasing, *PI(S;C*) shows how much the class entropy *H(C*) is reduced, when information about SWA becomes available under imperfect modeling. A positive value of *PI*(S;*C*) indicates a sound SWA model that should lead to the successful classification of a mouse with unknown age/exercise. Note that the computation of PI requires the estimated model P^r⁢ model(S =s|C =c), as well as the true distribution *Pr*_true_(*S* = *s*|*C* = *c*) of SWA, which is unknown and can only be sampled directly from the test dataset. Thus, the PI takes into account the model assumption and estimation errors that are introduced by the multivariate normal assumption and the respective parameter estimation (Durvaux et al., 2014). If the true and the estimated distribution are very close, then the PI metric is equivalent to classic mutual information. The PI metric was computed as follows:

• PI(S;C)=H(C)−H(C|S)=H(C)+Σcϵ𝒞nPr⁡[c]Σsϵ𝒟tcnPrtrue[s|c]⋅log⁡2(P^rmod⁡el[c|s])⁡
• P^rmodel[c|s]=P^rmodel[s|c]Σc*ϵ𝒞P^rmodel[s|c*] using the Bayes’ rule and P^r⁢ model[s|c] is estimated from ${𝒟}_{p}^{c}$
• ${\mathrm{Pr}}_{true}[s|c]=\frac{1}{n_{t}^{c}},$ where $n_{t}^{c}$ is the number of signals in test dataset ${𝒟}_{t}^{c}$, i.e., it is sampled from ${𝒟}_{t}^{c}$

To verify that our SWA classification procedure is accurate, we made use of the cross-validation technique (Efron and Tibshirani, 1993), which estimates how well our profiled model performs in practice. In particular, we applied a ‘leave-one-out’ strategy, which is justified by the limited number of samples available in our experiments. The cross-validation was performed as follows:

(1) The dataset 𝒟 is split to |𝒟| non-overlapping sets 𝒟 (*i*), *i* = 1,..,|𝒟| , each containing a single SWA signal that originates from a single mouse.
(2) For certain index *i* we defined a test set 𝒟_t_ = 𝒟(i) and profile set

$${𝒟}_{p}=U_{j\neq i}𝒟\left(j\right)$$

Thus, we selected a single SWA signal denoted by index *i* for testing and used the rest for profiling. The set 𝒟_p_ was further partitioned to the corresponding class profile sets ${𝒟}_{p}^{c}$, *c* = 1,…,|𝒞| , i.e., we used it to create profile sets for all groups.
(3) For the given partitioning (step 2), we computed *PI(S;C)* where P^r⁢ model[s|c] was estimated from ${𝒟}_{p}^{c}$ and Pr_true_[*s*|*c*]sampled from 𝒟_t_.
(4) Steps 2 and 3 were repeated for all possible partitions of 𝒟 to profile and test datasets, which in the case of ‘leave-one-out’ strategy is |𝒟| partitions. The PI estimates for every partition of step 2 were averaged and their variance showed the accuracy of the results.

We completed the PI approach by computing a confidence interval for the PI estimates. Specifically, we used a 10% bootstrap confidence interval (Efron and Tibshirani, 1993), where we resampled 100 out of all the computed PI values (step 4), sorted them and set the confidence interval endpoints at the 5 and 95% percentiles.

#### Success Rate

The success rate metric quantified how successful our profiled model (template) was in recognizing the age/exercise class of mice that were not part of the profiling group (${𝒟}_{p}^{c}$, thus expressing the classification’s accuracy as a percentage). We employed the success rate metric to confirm that a positive PI also leads to a correct classification. For every class *c* ∈ 𝒞, we partitioned again *D*^c^ to profile and test datasets ${𝒟}_{p}^{c}$ and ${𝒟}_{t}^{c}$, this time using a 50–50 ratio, instead of the ‘leave-one-out’ strategy. We split the dataset (𝒟^c^) in two roughly equal subsets, in order to show that even fairly small profiling sets (just 50% of 𝒟^c^) can provide accurate classification and high success rate. The profile datasets ${𝒟}_{p}^{c}$, *c* ∈ 𝒞 were used to estimate |𝒞| SWA templates P^r⁢ model[S|c]. Consequently, SWA signals from all test datasets ${𝒟}_{t}^{c}$, *c* ∈ 𝒞 were compared with the templates using the maximum likelihood principle to find the class *c* that is the best match. Thus, for all classes *c* ∈ *C* and corresponding signals ∈ ${𝒟}_{t}^{c}$, we computed *argmax*_c_P^r⁢ model[s|c]. Matching a signal from set ${𝒟}_{t}^{c}$ with any template other than the template of *c*, constituted misclassification, that was reflected on the success rate.

#### Unsupervised Learning Approach

Note that the class labels assigned to mice, i.e., age, exercise, are closely coupled with our experimental design and hypotheses; we implicitly assumed 6 distinct mouse groups and tried to distinguish between them. However, this artificial separation into groups may not be in accordance with the information present in the dataset, e.g., certain groups may present strong similarities. To investigate this, we expanded our analysis with unsupervised learning approaches that take out all the assumptions of the researcher and provide an unbiased look at the data. In other words, this approach can find the most reasonable clustering in the data without using prior labeling and gauge the similarities between different clusters.

In order to estimate the optimal number of clusters in the SWA data, we used the “Gap” statistic approach (Tibshirani et al., 2001). In this method, each cluster has a central point, named the centroid, and *k*-means clustering was used to compute the distances between the centroids of the clusters that were formed. The distance between the centroids is an indication of the similarity of the SWA characteristic features between the clusters.

To be precise, a number (*k*) of observations was randomly chosen as being prototypes for the clusters. Then, using the Euclidean distance function, the SWA patterns for all experiments (after PCA-based dimensionality reduction, from twelve to three dimensions) were appointed to the cluster where the distance function was minimal. Subsequently, the centroid was calculated for each cluster. Then individual samples were re-appointed to another cluster and the centroids were re-calculated. The procedure was repeated 1000 times to reach convergent behavior.

The Gap statistic approach uses first the result of *k-*means algorithm to compute the within-cluster sum of square around the cluster means. Second, this is compared to a reference distribution that is sampled from the dataset, which in turn leads to a score *Gap*[*k*]. *Gap*[*k*] score computation is used in order to evaluate the optimal number of clusters The *k-*means algorithm and the *Gap*[*k*]score computation are repeated for *k* = 1 until 6 clusters and the number of clusters *k* that optimizes the score is chosen.

## Results

### Running Wheel: Behavioral Data

Examples of 28 days running wheel activity from a 6-month, an 18-month and a 24-month old mouse are shown in **Figure 1B**. Young mice showed significantly increased average daily running wheel activity compared to aged mice (*6 months old:* 27746 ± 1974 counts/24 h, *18 months old:* 5190 ± 2521 counts/24 h, *24-months old:* 6458 ± 1884 counts/24 h) [One-way ANOVA, factor ‘exercise,’ *F*(2,23) = 34.91; *p* < 0.0001, *post hoc* Bonferroni tests: *p* < 0.0001]. Additionally, aging reduced significantly the strength of the 24-h rhythm (*6 months old:* 0.47 ± 0.02, *18 months old:* 0.16 ± 0.05, *24-months old:* 0.22 ± 0.05) [one-way ANOVA, factor ‘strength,’ *F*(2,23) = 16; *p* < 0.0001, *post hoc* Bonferroni tests: *p* < 0.001].

### Sleep Architecture

**FIGURE 2:**
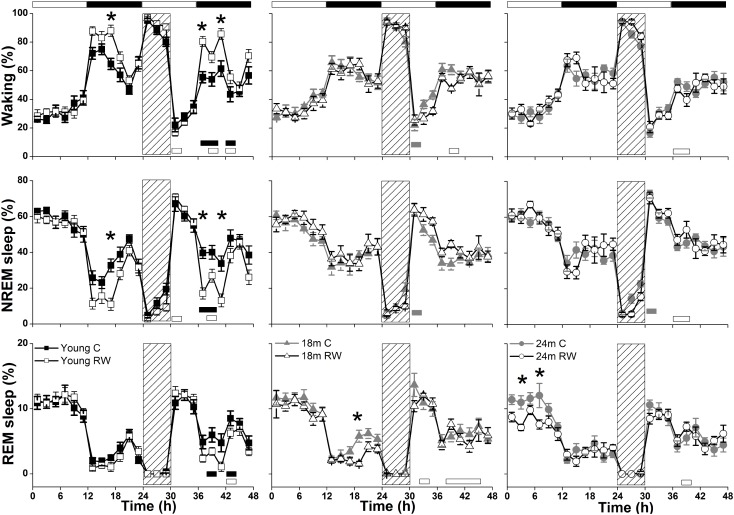
Time course of vigilance states, for 24-h baseline (BL), 6-h sleep deprivation (SD, hatched bar) and 18-h recovery for 6, 18, and 24 months old control and running-wheel (RW) mice (**left, middle, and right**, respectively). Curves connect 2-h mean values (±SEM) of Waking, NREM and REM sleep. The black and white bars above each graph indicate the light-dark cycle. Asterisks at the top of each graph represent significant differences between control and wheel access groups across the 48-h period and bars at the bottom of each graph significant differences between recovery and BL day for each age and condition (*post hoc* unpaired and paired *t*-tests with Bonferroni multiple comparisons correction, *p* < 0.05 after significant ANOVAs, main effects ‘treatment,’ ‘time of day,’ ‘day’).

During undisturbed BL, young mice that had access to the running wheel prior to the sleep recordings, showed increased waking and decreased NREM sleep in the first 6-h of the dark period, as compared to age-matched controls (**Figure 2**) (*post hoc* two-tailed unpaired *t*-tests between groups with Bonferroni multiple comparisons correction following significant interaction and main effects ‘treatment,’ ‘time of day’ and ‘day’) (statistical analysis can be found in **Table 1**). The latter was also evident after SD. No effects were found in REM sleep. A more moderate effect of regular activity was evident in the older mice, that was reflected in a reduction in BL REM sleep at the end of the dark period in the 18 months old mice and during the light period in the 24 months old mice (**Table 1**). SD did not result in additional vigilance state alterations in the young group. This was also the case for the 24 months old mice (**Table 1**). In the 18 months old mice that had the running wheel, the ANOVA showed a stronger increase in NREM sleep and decrease in waking, compared to age-matched controls, however, these effects did not survive *post hoc* multiple comparisons correction (**Table 1**). Compared to BL, the period after SD was associated with increased NREM sleep and REM sleep and reduced waking in all six groups (paired *t*-tests with Bonferroni multiple comparisons correction, *p* < 0.05 after aforementioned significant ANOVAs reported in **Table 1**).

**Table 1:** Statistical analysis.

Figure/Table	Vigilance state	Two- or three-way ANOVA	
		
		*Interaction factors*	*Main factors*
**Figure 2** (Young mice)	Waking	‘treatment^∗^day^∗^time of day’	‘treatment’ *p* < 0.0001 **^∗^**
		*F*(11,432) = 0.769	‘day’ *p* < 0.0001 **^∗^**
		*p* = 0.671 with ‘treatment^∗^time of day’	‘time of day’ *p* < 0.0001 **^∗^**
		*p* < 0.0001 **^∗^**	
	NREM sleep	‘treatment^∗^day^∗^time of day’	‘treatment’ *p* < 0.0001 **^∗^**
		*F*(11,432) = 0.782	‘day’ *p* < 0.0001 **^∗^**
		*p* = 0.658 with ‘treatment^∗^time of day’	‘time of day’ *p* < 0.0001 **^∗^**
		*p* < 0.0001 **^∗^**	
	REM sleep	‘treatment^∗^day^∗^time of day’	‘treatment’ *p* = 0.056
		*F*(11,432) = 0.527	‘day’ *p* < 0.0001 **^∗^**
		*p* = 0.885 with ‘treatment^∗^time of day’ p = 0.032 **^∗^**	‘time of day’ *p* < 0.0001 **^∗^**
**Figure 2** (18 months old mice)	Waking	‘treatment^∗^day^∗^time of day’	‘treatment’ *p* = 0.026**^∗^**
		*F*(11,360) = 0.898	‘day’ *p* < 0.0001 **^∗^**
		*p* = 0.542	‘time of day’ *p* < 0.0001 **^∗^**
	NREM sleep	‘treatment^∗^day^∗^time of day’	‘treatment’ *p* = 0.003 **^∗^**
		*F*(11,360) = 0.821	‘day’ *p* < 0.0001 **^∗^**
		*p* = 0.619	‘time of day’ *p* < 0.0001 **^∗^**
	REM sleep	‘treatment^∗^day^∗^time of day’	‘treatment’ *p* = 0.032 **^∗^**
		*F*(11,360) = 0.565	‘day’ *p* < 0.0001 **^∗^**
		*p* = 0.857	‘time of day’ *p* < 0.0001 **^∗^**
**Figure 2** (24 months old mice)	Waking	‘treatment^∗^day^∗^time of day’	‘treatment’ *p* = 0.825
		*F*(11,354) = 1.722	‘day’ *p* < 0.0001 **^∗^**
		*p* = 0.067	‘time of day’ *p* < 0.0001 **^∗^**
	NREM sleep	‘treatment^∗^day^∗^time of day’	‘treatment’ *p* = 0.726
		*F*(11,354) = 2.041	‘day’ *p* < 0.0001 **^∗^**
		*p* = 0.024 **^∗^**	‘time of day’ *p* < 0.0001 **^∗^**
	REM sleep	‘treatment^∗^day^∗^time of day’	‘treatment’ *p* = 0.02 **^∗^**
		*F*(11,354) = 0.741	‘day’ *p* < 0.0001 **^∗^**
		*p* = 0.699 with ‘treatment^∗^time of day’	‘time of day’ *p* < 0.0001 **^∗^**
		*p* = 0.008 **^∗^** and ‘treatment^∗^day’	
		*p* = 0.005 **^∗^**	
**Table 2** (Young mice)	Waking	‘treatment^∗^Light-Dark’ *F*(5,96) = 4.773	‘treatment’ *p* = 0.008 **^∗^**
		*p* = 0.001 **^∗^**	‘Light-Dark’ *p* < 0.0001 **^∗^**
	NREM sleep	‘treatment^∗^Light-Dark’ *F*(5,96) = 4.1	‘treatment’ *p* = 0.002 **^∗^**
		*p* = 0.002 **^∗^**	‘Light-Dark’ *p* < 0.0001 **^∗^**
**Table 2** (24 months old mice)	REM sleep	‘treatment^∗^Light-Dark’ *F*(5,96) = 3.8	‘treatment’ *p* = 0.007 **^∗^**
		*p* = 0.004 **^∗^**	‘Light-Dark’ *p* < 0.0001 **^∗^**
**Figure 3** (Young mice)	Waking	‘treatment^∗^frequency bin’ *F*(29,540) = 8.23	‘treatment’ *p* < 0.0001 **^∗^**
		*p* < 0.0001 **^∗^**	‘frequency bin’ *p* < 0.0001 **^∗^**
	NREM sleep	‘treatment^∗^frequency bin’ *F*(29,540) = 9.778	‘treatment’ *p* < 0.0001 **^∗^**
		*p* < 0.0001 **^∗^**	‘frequency bin’ *p* < 0.0001 **^∗^**
	REM sleep	‘treatment^∗^frequency bin’ *F*(29,540) = 3.949	‘treatment’ *p* = 0.0061 **^∗^**
		*p* < 0.0001 **^∗^**	‘frequency bin’ *p* < 0.0001 **^∗^**
**Figure 3** (18 months old mice)	Waking	‘treatment^∗^frequency bin’ *F*(29,450) = 2.42	‘treatment’ *p* < 0.0001 **^∗^**
		*p* < 0.0001 **^∗^**	‘frequency bin’ *p* < 0.0001 **^∗^**
	NREM sleep	‘treatment^∗^frequency bin’ *F*(29,450) = 1.173	‘treatment’ *p* < 0.0001 **^∗^**
		*p* = 0.248	‘frequency bin’ *p* < 0.0001 **^∗^**
	REM sleep	‘treatment^∗^frequency bin’ *F*(29,450) = 1.79	‘treatment’ *p* < 0.0001 **^∗^**
		*p* = 0.008 **^∗^**	‘frequency bin’ *p* < 0.0001 **^∗^**
**Figure 3** (24 months old mice)	NREM sleep	‘treatment^∗^frequency bin’ *F*(29,450) = 1.014	‘treatment’ *p* < 0.0001 **^∗^**
		*p* = 0.448	‘frequency bin’ *p* < 0.0001 **^∗^**
	REM sleep	‘treatment^∗^frequency bin’ *F*(29,450) = 1.109	‘treatment’ *p* < 0.0001 **^∗^**
		*p* = 0.32	‘frequency bin’ *p* < 0.0001 **^∗^**
**Figure 4** (all groups)	Slow-wave activity (SWA) in NREM sleep	‘treatment^∗^time of day^∗^day’	‘treatment’ *p* < 0.0001 **^∗^**
		*F*(40,988) = 0.03	time of day’ *p* = 0.124
		*p* > 0.99	‘day’ *p* = 0.377
**Figure 4** (Young control mice)	Slow-wave activity (SWA) in NREM sleep	‘time of day^∗^day’ *F*(8,89) = 11.05	‘day’ *p* = 0.05
		*p* < 0.0001 **^∗^**	‘time of day’ *p* = 0.93
**Figure 4** (Young RW mice)	Slow-wave activity (SWA) in NREM sleep	‘time of day^∗^day’ *F*(8,62) = 7.443	‘day’ *p* = 0.26
		*p* < 0.0001 **^∗^**	‘time of day’ *p* = 0.15
**Figure 4** (18 months old control mice)	Slow-wave activity (SWA) in NREM sleep	‘time of day^∗^day’ *F*(8,63) = 5.48	‘day’ *p* < 0.0001 **^∗^**
		*p* < 0.0001 **^∗^**	‘time of day’ *p* = 0.9995
**Figure 4** (18 months old RW mice)	Slow-wave activity (SWA) in NREM sleep	‘time of day^∗^day’ *F*(8,72) = 14.65	‘day’ *p* = 0.275
		*p* < 0.0001 **^∗^**	‘time of day’ *p* = 0.69
**Figure 4** (24 months old control mice)	Slow-wave activity (SWA) in NREM sleep	‘time of day^∗^day’ *F*(8,66) = 9.46	‘day’ *p* < 0.0001 **^∗^**
		*p* < 0.0001 **^∗^**	‘time of day’ *p* = 0.9988
**Figure 4** (24 months old RW mice)	Slow-wave activity (SWA) in NREM sleep	‘time of day^∗^day’ *F*(8,63) = 5.35	‘day’ *p* = 0.82
		*p* < 0.0001 **^∗^**	‘time of day’ *p* > 0.999


*Statistical analysis: two or three-way analysis of variance (ANOVA) was conducted to test the effect of exercise (treatment) and sleep deprivation (see text for more details). Asterisks following reported p values indicate significance (**^∗^***p* < 0.05).*

Young 6-month old mice were more awake during the dark period o older mice (**Table 2**). In addition, young exercising mice were even more awake and had less NREM sleep compared to young control mice (**Table 1**) (*post hoc* unpaired *t*-tests between groups in specific light-dark periods: *p* ≤ 0.05). The amount of REM sleep gradually decreased in the BL light period as a function of aging in the groups provided with a running wheel, where the 24 months old mice that had access to a wheel had the lowest amount of REM sleep in the BL light period among all groups (**Table 1**) (*post hoc* unpaired *t*-tests between groups in specific light-dark periods: *p* ≤ 0.05).

**Table 2:** 12-h Vigilance states.

	Waking		NREMs		REMs	
			
Light-Dark	L1	D1	L1	D1	L1	D1
Young RW	3.85 (0.21)	8.92 (0.18) **^∗^**	6.81 (0.2)	2.78 (0.2) **^∗^**	1.34 (0.05)	0.3 (0.02)
Young C	3.8 (0.33)	7.62 (0.25)	6.92 (0.23)	4.01 (0.22)	1.28 (0.09)	0.37 (0.04)
18 months RW	3.96 (0.27)	7.02 (0.36)	6.79 (0.3)	4.65 (0.35)	1.25 (0.07)	0.34 (0.03)
18 months C	4.23 (0.2)	7.08 (0.38)	6.46 (0.23)	4.41 (0.36)	1.31 (0.07)	0.52 (0.05)
24 months RW	4.02 (0.15)	6.9 (0.4)	7.03 (0.17)	4.7 (0.35)	0.95 (0.03) **^∗^**	0.39 (0.07)
24 months C	3.87 (0.22)	6.96 (0.33)	6.87 (0.19)	4.64 (0.31)	1.26 (0.06)**^∗^**	0.39 (0.04)


*Mean values (±SEM) in hours of Waking, NREM and REM sleep for 12-h light (L1) and dark (D1) of the baseline (BL) day. Black asterisks indicate significant difference between the group and all the others, and gray asterisk between the same age group (^∗^*p* ≤ 0.05).*

### EEG Power Density

**FIGURE 3:**
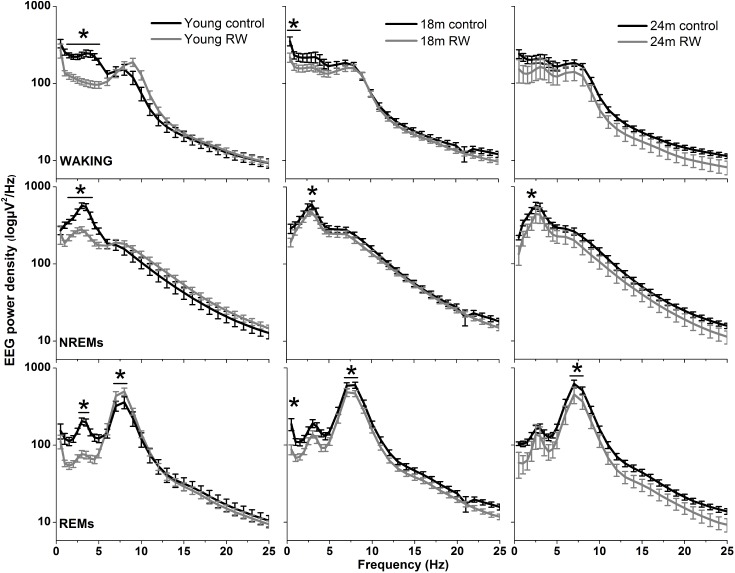
Absolute electroencephalographic (EEG) power density in Waking, NREM and REM sleep for 6, 18, and 24 months old control and running-wheel (RW) mice (**left, middle, and right**, respectively). Asterisks indicate significant differences between the control and wheel access groups (*post hoc* unpaired *t*-tests with Bonferroni multiple comparisons correction, *p* < 0.05 after significant ANOVA, main effects ‘treatment,’ ‘frequency bin’).

The absolute EEG power density spectrum was computed for the three vigilance states in all groups (**Figure 3**). The most pronounced differences in the waking and NREM sleep spectra were found in the young mice, where previous access to a running wheel decreased absolute EEG power density values in the slow frequencies (1–5 Hz) in waking and NREM sleep (*post hoc* two-tailed unpaired *t*-tests between groups with Bonferroni multiple comparisons correction following significant interaction and main effects ‘treatment’ and ‘frequency bin’) (**Table 1**). Similar decreases in the slow wave range, albeit less robust, were found in the 18 months old mice in waking and NREM sleep and in 24 months old mice in NREM sleep (**Table 1**). Notably, groups previously provided with a wheel demonstrated decreases in absolute power density values in the slow EEG frequencies during REM sleep (0.5–5.5 Hz), albeit not always surviving the multiple comparisons correction. Theta activity (7–8 Hz) was increased in the young exercising animals, but decreased in the older age groups who had a wheel, compared to age matched controls (**Table 1**).

### Absolute NREM and REM Sleep SWA

**FIGURE 4:**
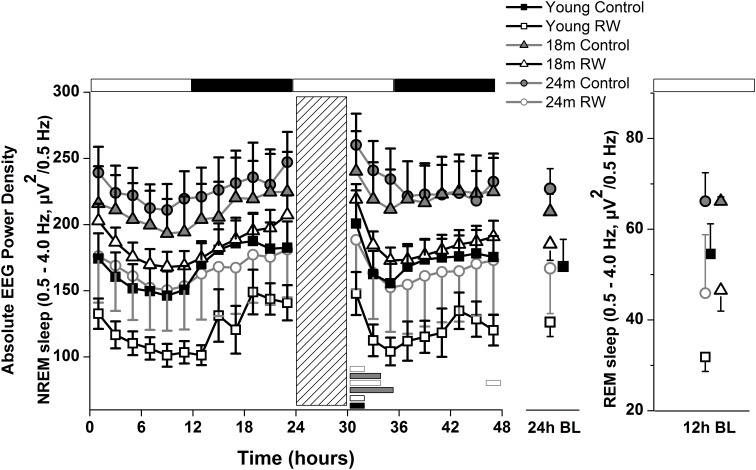
Time course of absolute electroencephalographic (EEG) power for the slow-wave activity range (SWA, 0.5–4 Hz) in non-rapid-eye movement (NREM) sleep for 24-h baseline (BL), 6-h sleep deprivation (SD, hatched bar) and 18-h recovery for control and running-wheel (RW) mice **(left)**, 24-h baseline (BL) EEG SWA data in NREM sleep (middle) and 12-h BL EEG SWA in REM sleep **(right)**. EEG SWA in NREM increased as a function of aging, and decreased with the use of a running wheel. Bars at the bottom of each graph significant differences between recovery and BL day for each age and condition (*post hoc* Bonferroni multiple comparisons correction *t*-tests, *p* < 0.05 after significant ANOVA, main effects ‘group,’ ‘treatment,’ ‘time of day,’ ‘day’).

To investigate whether the increase in SWA observed in aging could be diminished by the daily use of a running wheel, we computed the time course of absolute NREM sleep SWA in all groups (**Figure 4**). We found that EEG SWA in NREM sleep increased as a function of aging, and decreased with the use of the running wheel throughout the 48-h of recordings (**Table 1**) with interesting differences along the first 12-h of the light period where SWA is more pronounced in NREM sleep [One-way ANOVA, factor ‘group,’ *F*(5,66) = 90.17; *p* < 0.0001; *post hoc* Bonferroni tests: *p* < 0.05]. Notably, NREM sleep SWA levels of young control mice did not differ from the 18 and 24 months old RW mice (*post hoc* Bonferroni tests: *p* = 0.062 and *p* = 0.999 respectively). The two aged control groups and the two aged RW groups differed significantly (*post hoc* Bonferroni tests: *p* = 0.045 and *p* = 0.018 respectively). For clarity, we also plot the 24-h average BL EEG SWA in NREM sleep values and 12-h average BL light period REM sleep SWA values next to the EEG SWA in NREM sleep time course. The decrease in absolute SWA in NREM sleep levels in the RW groups, compared to age-matched controls, was the largest in the 24-months old mice (60.44 μV^2^/0.5 Hz), followed by the young mice (47 μV^2^/0.5 Hz) and smallest in the 18-months old mice (24.8 μV^2^/0.5 Hz). In all age groups the decrease was found to be significant [*F*(2,33) = 82.68; *p* < 0.0001; *post hoc* Bonferroni tests: *p* < 0.0001]. SD induced alterations in SWA in all groups, particularly an increase was evident in the first 2–6 h of the recovery period (**Table 1**).

### Profiling and Classifying Slow-Wave Activity (SWA)

#### Supervised Learning Approach

**FIGURE 5:**
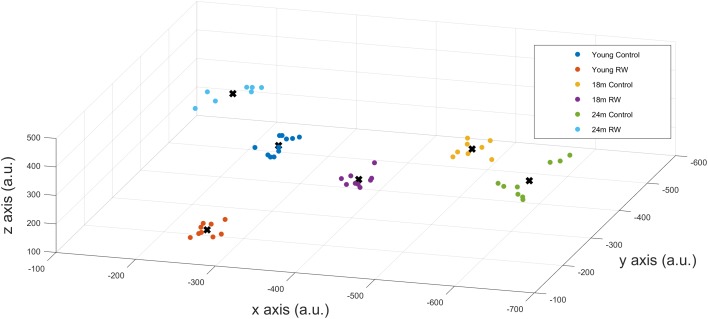
x-,y-,z- coordinates of the optimal number of clusters after PCA dimensionality reduction. Scatter plots represent the six experimental groups together with the central points (centroids) (see text for more details).

A positive PI was obtained with PI = 2.585 > 0 confirming that SWA in NREM sleep encloses information about age and exercise levels of the mice. Additionally, we could correctly classify mice that were not in the profiling groups (${𝒟}_{p}^{c}$) to the corresponding age and exercise group, confirming that the available information can be used to detect previously unknown details of the exercise and age of a mouse. We also computed the success rate of our classification technique which was found to be 100%. This means that we could accurately distinguish between the six groups and assign mice to the appropriate groups, according to their SWA levels, with maximum success rate. By performing dimensionality reduction with PCA we could plot the six different classes in a three dimension (3-D) figure (**Figure 5**).

#### Unsupervised Learning Approach

In order to detect similarity in SWA data between the different classes, we used Gap statistic clustering to find the optimal number of clusters from the SWA data. The optimal number of clusters was found to be six, indicating that it is equal to the number of classes. The centroids found for each cluster are plotted in **Figure 5** and the coordinates are given in **Table 3**. The distance between the clusters, as determined by the x, y, z coordinates of their centroids is an indication of similarity in SWA characteristics of the clusters (**Table 4**). Closer distances to the centroid of the young exercising mice were obtained for young control as well as 18 and 24 months old groups that had a wheel (Euclidean distance: ||*D*||_2_ = 222,267,344 respectively). Larger distances were found for the 18 and 24 months old control groups (Euclidean distance: ||*D*||_2_ = 385 and 430 respectively) (**Table 4**).

**Table 3:** Coordinates of the centroids.

	*x*	*y*	*z*
Young C	-290.4239343	-351.278	278.2895
Young RW	-258.3819842	-188.679	131.1928
18 months C	-480.2993301	-500.791	166.7696
18 months RW	-439.3514277	-214.311	326.1029
24 months C	-636.4445877	-261.612	322.952
24 months RW	-221.3199115	-384.299	412.169


**Table 4:** Euclidian distances between the centroids.

	Young C	Young RW	18 months C	18 months RW	24 months C	24 months RW
**Young C**	0.00	221.59	266.16	207.91	360.23	154.24
**Young RW**	221.59	0.00	384.61	267.20	430.14	344.37
**18 months C**	266.16	384.61	0.00	330.36	325.55	375.32
**18 months RW**	207.91	267.20	330.36	0.00	202.71	289.55
**24 months C**	360.23	430.14	325.55	202.71	0.00	441.97
**24 months RW**	154.24	344.37	375.32	289.55	441.97	0.00


*Euclidian distances based on the (*x, y, z* coordinates) values in **Table 3**.*

## Discussion

In this study, we show that long-lasting voluntary physical activity in mice, corresponding to regular exercise in humans, alters EEG SWA throughout the whole age spectrum. EEG SWA is implicated in sleep homeostasis and constitutes an important parameter that merits observation. Analytically, our experimental data demonstrate that EEG SWA decreased with exercise and increased with aging. The young mice that were provided with a running wheel had the lowest SWA levels, whereas the oldest sedentary mice had the highest. Notably, the effect of long-term voluntary exercise was sustained for almost 2 weeks after removal of the running wheel. With regards to sleep architecture, the exercise effect was particularly pronounced in young mice, yet appeared to be attenuated in the older mice. Interestingly, our data show that introduction of moderate exercise even later in life leads concretely to a younger brain phenotype; this is mainly reflected in the NREM sleep EEG SWA. Due to the prominent and expository nature of EEG SWA, we conducted additional analysis using information-theoretic and machine-learning approaches. These methods were able to extract information for age and previous opportunity of physical activity of the mice, and, with cluster analysis, we could classify and accurately distinguish the different groups based solely on their EEG SWA. Thus, our conclusions are three-fold, first, effects of exercise on sleep persist following 2 weeks after removal of the wheel, second, we suggest that EEG SWA can be used as a physiological marker of brain age in the mouse and, third, we show that exercise can lead to a younger brain phenotype.

### NREM Sleep EEG SWA: Physiological Marker of Brain Age

Slow-wave-activity levels increases in the course of aging in mice, likely denoting alterations in brain connectivity in the cortex (Panagiotou et al., 2017). In the current study, we introduced a running wheel for daily voluntary use and found that this condition diminishes the age-related increase in EEG SWA toward levels similar to young mice. The data show that EEG SWA in mice increases in the course of aging, regardless of wheel availability, and it decreases in all groups with voluntary exercise. Aged mice that were provided with a wheel and young control mice share very similar SWA characteristics, suggesting that the use of a running wheel alters EEG SWA toward a younger phenotype.

Since distinct characteristic SWA patterns were evident for each group, we conducted additional statistical analysis on EEG SWA data in order to investigate whether SWA can predict the age and previous opportunity of physical activity of the mice. We were able to distinguish groups and successfully predict the age and exercise condition, on the basis of their EEG SWA, with maximum success rate (100%). With cluster analysis based on the Gap statistic approach, we found that the optimal number of clusters was six, and the clusters coincided with the different experimental groups. This indicates that the characteristic features, that can be found in the SWA, by these clustering methods, are sufficiently different to distinguish meaningful groups from the SWA data. The distance between the clusters, as determined by their centroids, is an indication of similarity in SWA characteristics of the clusters.

**FIGURE 6:**
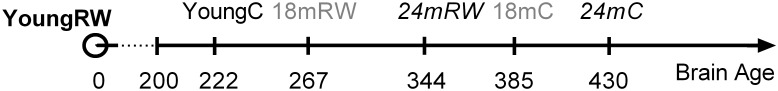
Suggested relative brain age of mice represented as the distances between the clusters’ centroids of young running-wheel (RW) mice (set as 0, YoungRW) and the other groups. The one-dimension (1-D) values are computed using the Euclidean distances from **Table 4** based on the 3-D values reported in **Table 3** for young, 18 and 24 months old control and 18 and 24 months RW mice. Hidden brain age is revealed in 18 and 24 months old mice, which is attenuated by 30 and 20% respectively, depicting a rejuvenating path following long-term physical activity.

Physical activity is thought to be beneficial for general health and, additionally in promoting healthy sleep cycles and healthy aging (Hillman et al., 2008; Kaliman et al., 2011; Hötting and Röder, 2013; Voss et al., 2013b), hence, we can consider the young RW mice to be the healthy control group. Therefore, we computed and compared the distances based on the x-, y- and z- coordinates between the young RW group and young, 18, 24 months control and 18 and 24 months RW groups (these are indicated in **Table 4**). We found that young control mice had the closest distance, whereas aged control mice revealed the largest one. Interestingly, aged RW mice were closer to the young RW mice as compared to aged controls. However, still these distances were larger than the distance between young RW mice and young controls. In addition, as a potential health indicator, we can consider the distance of the other clusters’ centroid to the centroid of the young RW mice, as a marker of a “brain age.” In other words, we know the chronological age of the mice (18 or 24 months old), and in addition their biological “brain age” which is reduced with physical activity by 30% in 18 months old mice and 20% in 24 months old mice (**Figure 6**). In this figure, the distance of the other clusters’ centroid to the centroid of the young RW mice, reported earlier in **Table 4**, is plotted as a 1-D data representation indicating a putative biological “brain age.” Thus, our data show that a long-term effect of exercise prevails in the homeostatic component of sleep, i.e., EEG SWA. This modulation is sustained for at least 2 weeks after exercise has ended. Therefore, we can affirm that the NREM sleep EEG SWA is a physiological marker of brain age in the mouse.

Recent studies demonstrated that SWA in NREM sleep was increased in the course of aging both in the local as well as in the global level (Panagiotou et al., 2017; McKillop et al., 2018), however, local cortical neural dynamics and local sleep homeostatic mechanisms, could remain largely unaffected, indicating that protective or compensatory mechanisms may exist to maintain neuronal function stable across the life span (McKillop et al., 2018). Our study suggests that global dynamics associated with age-induced alterations are mainly affected through regular physical activity leading to a potential ‘younger phenotype.’

In addition to a potential ameliorated brain function and/or brain age marker that we propose, SWA may reflect a generally improved physiology due to physical activity. Since regular exercise can prevent or delay diseases developed throughout life (Booth et al., 2012), SWA may be a “health marker” of the exercise effect in the course of aging, not only brain-specified but also body-specified. Although, in the present experiment we mainly analyzed a marker of brain activity and can only speculate about bodily health, generally the notion exists that a healthy body likely implies a healthy brain and vice versa, however, the pathways in which this may occur remain unknown. In addition thus to a younger brain phenotype, a younger body phenotype may emerge owing to body-brain interactions. Nevertheless, a beneficial effect of exercise is supported by our data displaying consistent results in the whole age spectrum regarding the brain and overall body health.

In contrast to mice, human EEG SWA generally tends to decrease in the course of aging. Following acute, as well as long-term exercise, SWA levels and slow-wave-sleep increased in old humans, reaching levels closer to younger subjects (Edinger et al., 1993; Horne, 2013; Chennaoui et al., 2015; Melancon et al., 2015). In old rats, analogously, 8-weeks of daily aerobic exercise led to altered sleep EEG characteristics that reached again levels closer to younger rats. Notably some of the effects were persisting for 2 weeks after the end of exercise (Blanco-Centurion and Shiromani, 2006). Our data corroborate this shift toward a younger phenotype due to exercise, i.e., EEG SWA shows an attenuated pattern in aged RW mice. We conjecture that, irrespective of the direction of the SWA change across species, physical activity makes SWA mimic younger patterns. Still, detailed EEG analysis including also SWA levels, that could reveal important insights regarding cortical brain characteristics (Riedner et al., 2011; Fogel et al., 2012; Mander et al., 2013), remains limited across human and animal research and we encourage further studies to solidify this conjecture.

Modest exercise is likely to be beneficial at all life stages, however, it has been proposed that early intervention might be important for a plethora of physiological features, such as cognitive health maintenance and improvement in the course of aging (Richards et al., 2003; Hillman et al., 2008). Our data show that interventions even later in life can also have positive effects on brain activity, such as on SWA levels. This was the case even when the amount of sleep did not change significantly in the aged mice. In elderly humans, the positive effects of physical exercise intervention later in life are evident for periods of weeks to months (Archer, 2011). For example, it was recently demonstrated that 1-year aerobic exercise improved markers of brain function in healthy older adults (Voss et al., 2013a). Thus, even in the case of later intervention, advantageous effects regarding brain function and consequently overall health are apparent, as shown in our study concerning the aged mice.

### Wheel Activity and Behavior

Running wheel activity is depicted as an elective behavior, not only in the laboratory, but also in the wild (Meijer and Robbers, 2014). It has been associated with enhanced cognitive development across the lifespan and, consequently, protection from age-related cognitive decline (Yang et al., 2012; Voss et al., 2013b). The importance of voluntary wheel-running, where animals are allowed to determine running time, speed, and distance, mimicking human choices, has been discussed earlier (Voss et al., 2013b). In the current study, we find that with voluntary daily use of a running wheel, the level of EEG SWA can be manipulated in all ages.

It has been suggested that the capacity to exercise is attenuated in the course of aging (Fleg, 1986; Silverman and Mazzeo, 1996). However, even low levels of physical activity, including regular daily activities, affect cognitive variables and neuroplasticity as shown in older adult humans (Ruscheweyh et al., 2011; Hötting and Röder, 2013). Neurogenesis is known to continue throughout life, but, although its level is attenuated considerably with age (Kuhn et al., 1996), physical activity increases cell proliferation rates even in older animals (Kronenberg et al., 2006). In a similar way, the aged mice in our study run significantly less compared to young ones; nevertheless, this is sufficient to alter sleep and EEG SWA, indicating the importance of age-matched exercise. We suggest that EEG SWA reveals a rejuvenating path in the aged mice, even with modest exercise levels, as evidenced in the reduced distance between the clusters of the young and/or RW groups of mice compared to aged controls. Previous studies showed that, an age-related deterioration of scale invariance in behavioral rest-activity fluctuations exists, where blocking the running wheel leads to a loss of scale invariance in both young and aged mice (Gu et al., 2015). Similar to detrended fluctuation analysis, a mathematical tool, being used to compute the scale invariance, EEG SWA can be defined as a physiological tool to determine brain health in the mouse.

### Sustained Effects of Regular Exercise on Sleep Architecture

The effect of running wheel availability on the sleep-wake cycle in the young mice persisted for at least 2 weeks after removal of the wheel. It consisted of increased waking and decreased NREM sleep, in the first part of the dark period, resembling the sleep patterns in mice that were recorded concomitantly with the running wheel in their cages (Lancel et al., 2003; Vyazovskiy et al., 2005). In the course of aging, this effect is diminished. In a similar way, biomarkers of brain activity and neurogenesis, such as BDNF protein levels were shown to be altered due to exercise in young (2 months), middle-aged (15 months) and old (24 months) animals. However, only young animals maintained a significant increase above baseline levels, after 2 or 4 weeks after the end of exposure to a running wheel (Adlard et al., 2005; Fischer et al., 2007; Van Praag, 2008), comparable to the changes found in the sleep architecture results in our study, showing that mainly young animals maintained significant sleep-wake alterations. Together, the data suggest that in the course of aging the ability to maintain sleep architectural effects induced by exercise is reduced. In an earlier study with forced exercise in rats (Blanco-Centurion and Shiromani, 2006), sleep architecture was ameliorated after 1-h exercise for 8-weeks, an effect that was sustained even 8-weeks after the exercise had ended. In contrast, the mice in our study were not forced but used the running wheel on a voluntary basis, which resulted in moderate levels of total exercise. The voluntary nature may explain why there is an improvement in sleep quality in the aged mice, when the wheel is available and used, and also why this improvement cannot be sustained for very long when the wheel is removed.

### Waking and REM Sleep EEG Power Density

The most pronounced effects of exercise on the EEG spectra in all the vigilance states were found in the young mice. The waking spectra showed decreased power in the low frequencies and increased power in the theta frequencies (around 9 Hz) which indicates increased activity and alertness in this group (Huber et al., 1999; Vyazovskiy et al., 2005).

Interestingly, a decrease in REM sleep slow-wave or delta power and low theta (∼0.5–5.5 Hz) and alterations in frequencies between 7 and 8 Hz were found in all age groups that were housed with a running wheel, compared to their age-matched controls. These EEG frequencies in REM sleep have been associated with hippocampal functioning (Bódizs et al., 2001). The hippocampus is an active region during REM sleep and its function seems to benefit from exercise (Dang-Vu et al., 2010; Horne, 2013). Additionally, it has been associated with spatial navigation, particularly in “cognitive map” creations (Roche et al., 2005; Andersen et al., 2007) and it is closely associated with the anterior cingulate cortex, a cognitive specific area which is also active during REM sleep (Dang-Vu et al., 2010). In the elderly, it was demonstrated that exercise could lead to increased connectivity between the hippocampus and the anterior cingulate cortex (Burdette et al., 2010). Furthermore, wheel running was shown to increase neurogenesis in the hippocampus, and to increase neuronal spine density, synaptic plasticity, neurotrophin levels, and spatial memory function in mice (Fischer et al., 2007; Van Praag, 2008). All these changes may influence REM sleep EEG spectra. Notably, the neuronal effects were considered to be correlated to epigenetic changes in the hippocampus and cerebral cortex and were shown to start within 3-h after wheel running and persist for 2 weeks (Fischer et al., 2007). Therefore, the consistent decrease of power density in the slow EEG frequencies during REM sleep over all age groups may reflect neuronal changes generated by long-term exercise. However, a difference was found between young and aged groups in frequencies between 7 and 8 Hz, in which young mice provided with a wheel showed an increase, whereas both aged groups showed a decrease, compared to age-matched controls. It was demonstrated earlier that, despite the fact that the rate of production of new cells in the dentate gyrus of the hippocampus was reduced in aged animals (Kuhn et al., 1996), a positive effect of physical activity was apparent on memory performance, neurogenesis and apoptosis (Kim et al., 2010). Thus, it seems that the intensity of exercise in combination with the increased age induces different changes in hippocampal brain connectivity evident in these spectra.

## Conclusion

Here, we show that sleep-wake rhythms and brain EEG variables are improved and that the level of the main sleep homeostatic component is significantly altered with moderate, age-matched exercise. We suggest that SWA is a physiological marker of brain age in the mouse, which emphasizes the importance of regular physical activity to promote and/or maintain physical and mental health. Physical activity, sleep as well as general health, declines in the course of aging. Daily exercise could attenuate the effects of sedentary behaviors, especially in older adults, and it could be prescribed as a first-order “medication” for general body and brain health augmentation. Future research directions could investigate human SWA patterns in order to extract age, exercise or other characteristics and validate the translational aspect of our findings.

## Data Accessibility Statement

The authors deposit supporting information and datafiles to Figshare for data archiving: https://figshare.com/s/97b6e1f60157d318318f.

## Author Contributions

TD and JM designed the research. MP and TD performed the research. MP, KP, JR, and TD analyzed the data. MP, KP, JR, JM, and TD wrote the paper.

## Conflict of Interest Statement

The authors declare that the research was conducted in the absence of any commercial or financial relationships that could be construed as a potential conflict of interest.
